# Ultra-Weak Fiber Bragg Grating Sensing Network Coated with Sensitive Material for Multi-Parameter Measurements

**DOI:** 10.3390/s17071509

**Published:** 2017-06-26

**Authors:** Wei Bai, Minghong Yang, Chenyuan Hu, Jixiang Dai, Xuexiang Zhong, Shuai Huang, Gaopeng Wang

**Affiliations:** 1 National Engineering Laboratory for Fiber Optic Sensing Technologies, Wuhan University of Technology, Wuhan 430070, China; weibai@whut.edu.cn (W.B.); daijixiang@whut.edu.cn (J.D.); xuexiangzhong@whut.edu.cn (X.Z.); huangshuai@whut.edu.cn (S.H.); w32136@whut.edu.cn (G.W.); 2 Key Laboratory of Fiber Optic Sensing Technology and Information Processing, Ministry of Education, Wuhan, 430070, China

**Keywords:** fiber optics sensors, relativity, multi-parameter

## Abstract

A multi-parameter measurement system based on ultra-weak fiber Bragg grating (UFBG) array with sensitive material was proposed and experimentally demonstrated. The UFBG array interrogation principle is time division multiplex technology with two semiconductor optical amplifiers as timing units. Experimental results showed that the performance of the proposed UFBG system is almost equal to that of traditional FBG, while the UFBG array system has obvious superiority with potential multiplexing ability for multi-point and multi-parameter measurement. The system experimented on a 144 UFBG array with the reflectivity of UFBG ~0.04% for the four target parameters: hydrogen, humidity, temperature and salinity. Moreover, a uniform solution was customized to divide the cross-sensitivity between temperature and other target parameters. It is expected that this scheme will be capable of handling thousands of multi-parameter sensors in a single fiber.

## 1. Introduction

The development trends towards the next generation of optical fiber sensing networks aim for huge capacity, long distance, and high precision. The huge capacity of optical fiber sensing networks includes two aspects of content, i.e. more points and more parameters of measurement. Jiang et al. proposed an on-line writing no-weld UFBG array through fiber drawing tower [1]. Then, Hu et al. demonstrated a single optical fiber network with over 1000 UFBG sensors by combining two semiconductor optical amplifiers (SOAs) and a time-division multiplexing (TDM) technique. Multi-point measurement based on UFBG array has made great improvement, while the multi-parameter measurement is still challenging [2,3].

In this paper, a serial TDM sensing network for multi-parameter measurement based on UFBGs coated with sensitive materials is proposed and experimentally demonstrated [4]. A UFBG array sensing system for hydrogen, relative humidity (RH), temperature and salinity measurements in a single fiber by coating different sensitive materials on different UFBGs is realized [5,6]. It can be concluded that thousands of UFBG sensors multiplexed in series with multi-parameter measurements could be possible, which shows promising applications for the future. 

## 2. Principle

### 2.1. Sensing with Coated FBG

**Figure 1:**
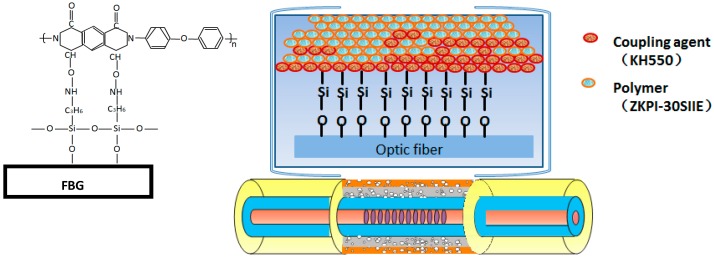
Structure of a coated FBG sensor.

When the FBG is subjected to external field effect (such as stress, temperature), the spatial period of grating will change, leading to the reflection or transmission grating central wavelength drift. Since they are immune to chemical parameters, such as humidity, hydrogen, salinity, etc., the FBG-based multi-parameter sensors are fabricated by coating corresponding sensitive materials on the surface of the fiber cladding covering the Bragg grating section. For instance, an RH-sensitive polymer is coated on the FBG fiber to measure RH, as shown in Figure 1.

When the RH and salinity variation changes, the volume expansion or volume contraction of the sensitive polymer coating will introduce mechanical strain on the FBG fiber, and therefore induce FBG central wavelength shift [7]. The measurement of the hydrogen concentration is based on the same principle, for which the sensor is covered with Pd. The accumulating central wavelength shift associated with multi-parameter (strain and temperature) measurement of FBG can be analytically calculated from Equation (1).

(1) $$\frac{\Delta \lambda_{B}}{\lambda_{B}}=\left(1-P_{e}\right)\cdot \beta \cdot \frac{(b^{2}-a^{2})\cdot Y_{C}}{a^{2}Y_{F}+(b^{2}-a^{2})\cdot Y_{C}}\cdot \Delta M+\left(\left(1-P_{e}\right)\cdot \frac{(b^{2}-a^{2})\cdot Y_{C}}{a^{2}Y_{F}+(b^{2}-a^{2})\cdot Y_{C}}\cdot \left(\alpha_{M}-\alpha \right)+\left(\alpha +\xi \right)\right)\cdot \Delta T$$

Where $\lambda_{B}$ is the central wavelength of the FBG; $P_{e}$ is the effective photo-elastic constant; $\alpha_{M}$ is the thermal expansion coefficient; β is an average expansion coefficient of the target parameter; ΔM is the normalized change of the target parameter; α and ξ are the thermal expansion coefficient and the thermo-optic coefficient of the single mode fiber, respectively; a and b are the cladding and total sensor diameters, respectively; $Y_{C}$ and $Y_{F}$ are the Young’s moduli of the coating materials and the silica fiber; and ΔT is the temperature change. For general silica fiber, $P_{e}$ ≈ 0.22, α ≈ 5.5 × 10^−7^ °C^−1^, ξ ≈ 6.67 × 10^−6^ °C^−1^ [8,9,10].

The desired parameter ΔM can be calculated by $\Delta \lambda_{B}$ and ΔT. $\Delta \lambda_{B}$ can be read by the central wavelength demodulation system. As FBG is inherently temperature sensitive, ΔT can be obtained and compensated by an FBG temperature sensor without any sensitive material coating. This is also the typical solution for the separation of strain and temperature cross-effect in mechanical parameter measurements.

### 2.2. Interrogation System

**Figure 2:**
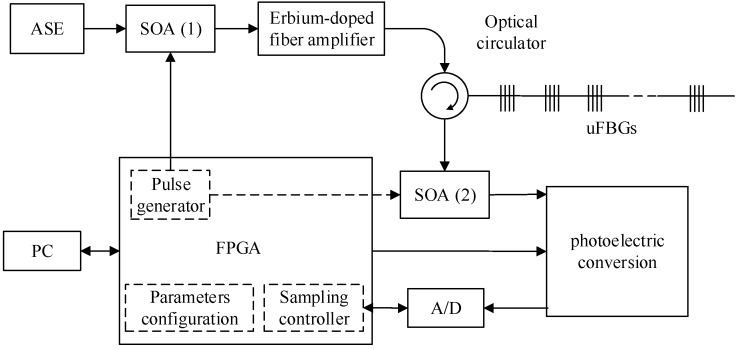
Schematic of the interrogation system.

A schematic of the multi-parameter sensing network interrogation system for identical UFBGs is illustrated in Figure 2. Field Programmable Gate Array (FPGA) takes the role of crucial scheduler of the central wavelength readout system, which includes three functions: (1) optical pulse controller for switching SOA1 and SOA2 on/off, (2) data acquiring controller for trigging the analog to digital (A/D) to sample the photoelectric conversing signals at a given time, and (3) communication with the personal computer (PC) for working environment configuration and data transition [11].

## 3. Experiment

### 3.1. Sensor Fabrication

The fabrication system for on-line writing FBG arrays during fiber drawing has been successfully developed in our laboratory; all of the 144 UFBGs are continuously sequenced along the fiber, the peak wavelengths are ~1552 nm, and the peak reflectivity is about 0.04%.

Without loss of generality, the first four UFBGs are chosen to measure hydrogen, RH, temperature, and salinity, respectively. Prior to the sensitive materials coating, the surface of the sections to be coated was cleaned by anhydrous alcohol and then the ultrasonic cleaner for 10 min under 30°C. Subsequently, the sensitive coatings were heat-treated at 85 °C for 20 min in thermostat. For the UFBG3 that was to be used for temperature sensing, no more treatment was necessary. 

**Figure 3:**
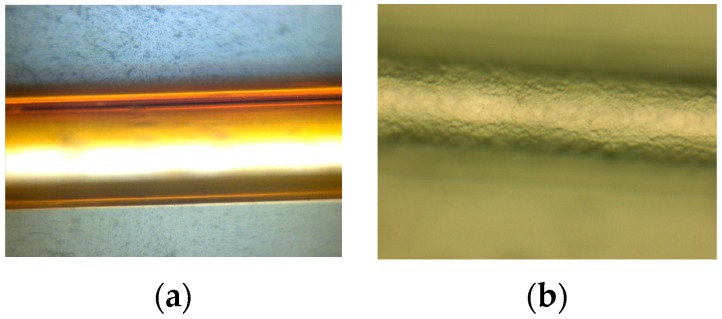
(**a**) Polyimide-covered UFBG; (**b**) Pd/Ni-covered UFBG.

Polyimide (ZKPI-305IIE, POME Sci-tech Co., Ltd., Beijing, China; solid content: 12~13%, viscosity: 5000–6000 cp) was chosen as the polymeric layer material because of its linear and reversible response to humidity change. UFBG2 and UFBG4 were fabricated as RH and salinity sensors, respectively, with ZKPI-305IIE polyimide as a sensitive coating. The UFBGs were first dip-coated with silane coupling agent (silane coupling agent: alcohol: deionized water = 20%:72%:8%) for 10 min to enhance the adhesion at the polymer interface. Second, the UFBGs were placed in a drying cabinet at 80 °C for 1 h, following which they were ready for the polymer layer coating. The fiber grating was dipped into the polyimide solution for 5–10 min and dried in a drying cabinet for a short thermal treatment at 150 °C. This process was repeated several times for the sake of obtaining the desired film thickness. With the purpose of fabricating a uniform polyimide film on the surface of the fiber, the rate of UFBG fiber rising-up and dropping-down were set at 600 um/min. Finally, the coating profile of the sensor was checked by an optical microscope, and as shown in Figure 3a, the film thickness of UFBG2 and UFBG4 are 14.1 μm and 13.4 μm, respectively [12].

Pd/Ni composite film is an ideal candidate for hydrogen sensors due to its durability, fast response, and relatively low cost. Pd/Ni composite film was sputtered on the etched UFBG1 by using a BESTECH sputtering system. First, a 10-nm Cr film was deposited on the side-face of the FBG as a basal layer by the radio-frequency (RF) sputtering process. Second, a 100-nm Pd/Ni composite film was sputtered on the etched FBG by a co-sputtering process. Under 0.5 Pa sputtering pressure of Ar, the deposition power for Pd and Ni targets are 100 and 50 W, respectively, which corresponds to a deposition rate of 0.14 and 0.01 nm/s, respectively. With this sputtering process, the atomic ratio of Pd and Ni is about 91:9 in Pd/Ni composite film. The thickness of the hydrogen- sensitive film was monitored by the quartz crystal method, which could ensure the thickness of the hydrogen-sensitive film as shown in Figure 3b [13,14]. 

### 3.2. Experimental Results

The experiment and interrogation system for multi-parameter measurement system is shown in Figure 2. During the initialization stage of the interrogation system, the delay timer for SOA1 and SOA2 was stepped 1 ns in every scanning period. Then, the local maximum of the reflected optical pulse spectrum from the InGaAs linear image detector was calculated, and the space positions of every UFBG were determined by Equation (1). 

**Figure 4:**
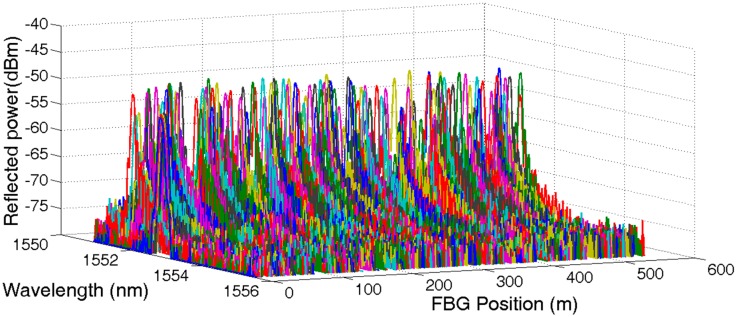
Reflective spectrum of low reflectivity UFBG array.

**Figure 5:**
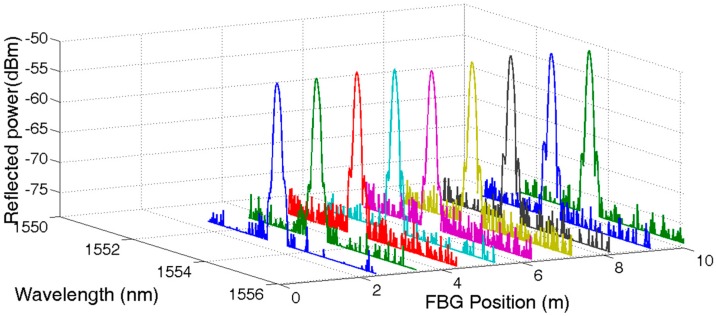
Reflective spectrum of UFBG1~UFBG9.

Setting the delay timer equal to 30 ns, 50 ns, 70 ns, and 90 ns, respectively, the reflected spectrum of UFBG1, UFBG2, UFBG3, and UFBG4 were obtained accordingly. All 144 reflected spectrums were normalized and are shown in Figure 4. The first nine signal spectrums were amplified partially for visible details and are shown in Figure 5.

#### 3.2.1. Temperature Measurement

**Figure 6:**
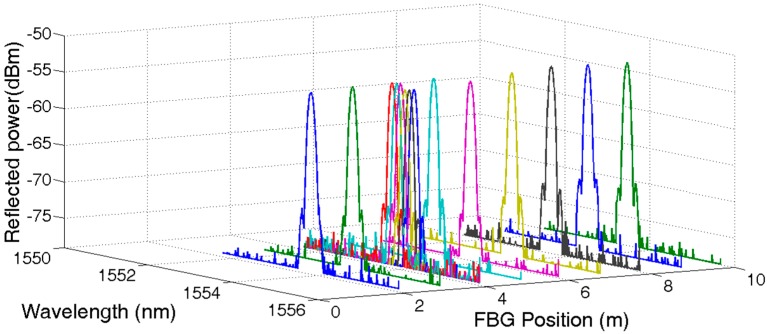
Reflective spectrum shift of UFBG3 during temperature measurement.

The temperate and humidity chamber model 101-0AB was used for the temperature and RH test. The adjusted temperature range of the chamber was limited to 20 °C–250 °C with ±1 °C at room temperature. UFBG3 was placed in the chamber while the other UFBGs were kept at 25 °C. The spectrum shifting of the first nine sensors due to the change in the chamber temperature is shown in Figure 6. The shape of the reflected spectrum of UFBG3 remained unchanged with the increase of temperature. The central wavelength can be could be calculated from Equation (1). As expected, apart from UFBG3, the reflected spectrum of the other UFBGs remained the same. 

**Figure 7:**
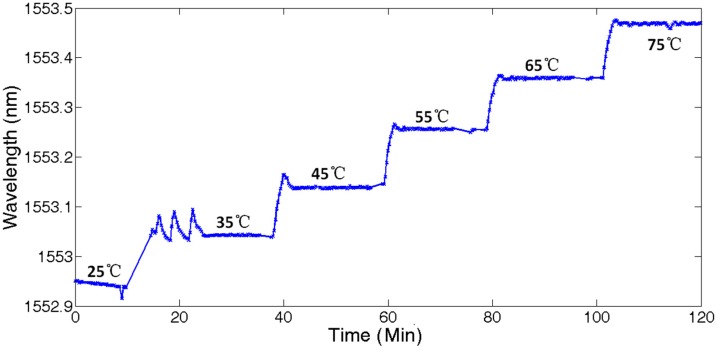
Temperature measurement of UFBG3.

Figure 7 shows the continuous 120 min temperature test results of UFBG3 by setting the chamber temperature at 25 °C, 35 °C, 45 °C, 55 °C, 65 °C, and 75 °C respectively (75% RH). It can be seen that the temperature response curve was homogeneous and stable. The jitter at 35 °C can be attributed to the first regulation fluctuation of the chamber temperature.

**Figure 8:**
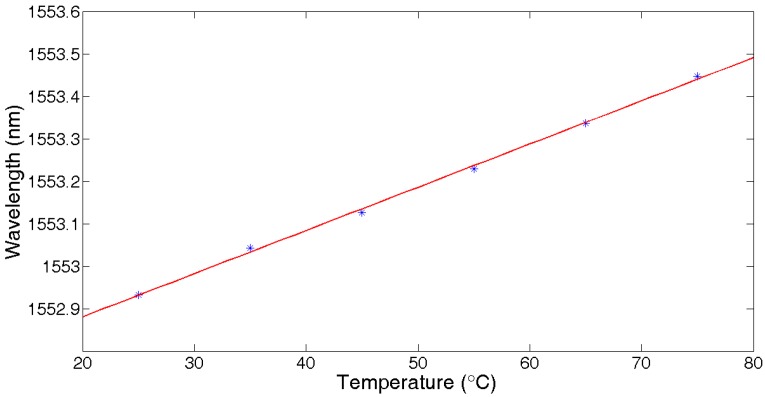
Central wavelength vs. temperature.

Figure 8 shows the linear fitting results of temperature with the central wavelength shift of UFBG3. The fitting function was f(x)=0.01074x+1552.677, so the temperature sensitivity was about 11 pm/°C.

#### 3.2.2. RH Measurement

**Figure 9:**
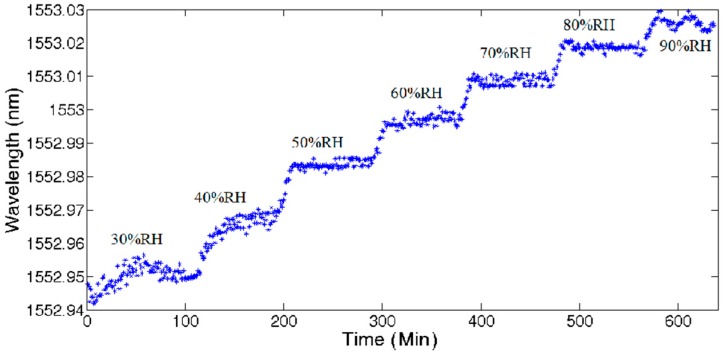
UFBG2 central wavelength evolution with the change of relative humidity.

UFBG2 was placed in the temperature and humidity chamber while the temperature was maintained at 30 °C. Keeping the temperature constant and changing the relative humidity from 30% RH to 40% RH, 50% RH, 60% RH, 70% RH, 80% RH, and 90% RH, respectively, the shift of the central wavelength was recorded over 600 min continually with an RH adjustment at about every 100 min as shown in Figure 9. The results verify the fact that the chamber presents unstable conditions at low or high RH settings while the temperature is fixed.

**Figure 10:**
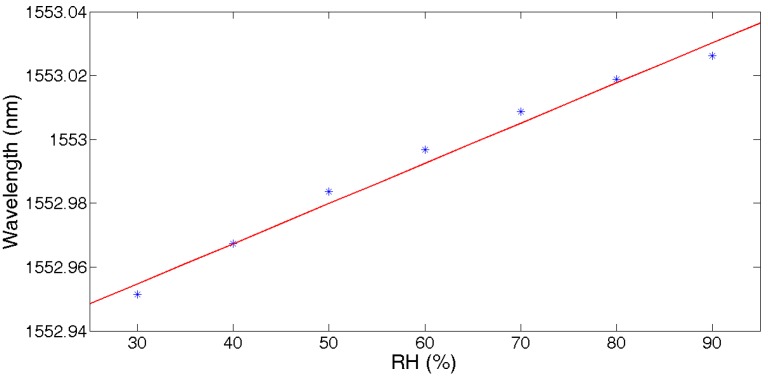
Central wavelength vs. humidity.

Figure 10 shows the fitting curve of the central wavelength with RH. The fitting function was f(x)=0.001258x+1552.917, and the RH sensitivity was about 1.26 pm/%RH. 

#### 3.2.3. Hydrogen Concentration Measurement

**Figure 11:**
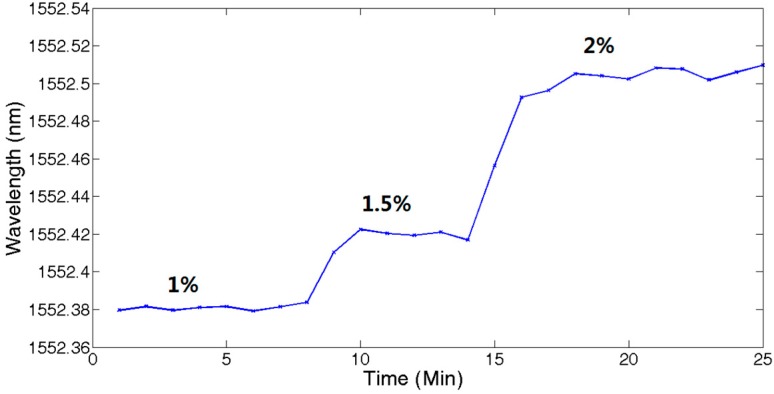
Hydrogen concentration measurement.

UFBG1 was place in a chamber with varying concentrations of hydrogen, while the other ambient parameters of the UFBGs were fixed. Figure 11 shows the central wavelength shift of UFBG1 with the concentrations of hydrogen at 1%, 1.5%, and 2%, respectively. 

**Figure 12:**
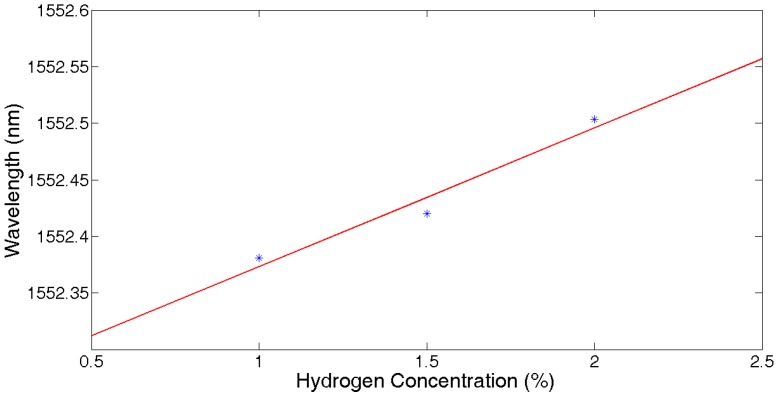
Central wavelength vs. hydrogen concentration.

The mean value of samples at 7~9 min at the same hydrogen concentration was calculated. Figure 12 shows the fitting function to be f(x)=0.115376x+1552.258. When hydrogen concentrations were from 1% to 1.5% and from 1.5% to 2%, the wavelength shifts of the FBG are 40 pm and 75 pm.

#### 3.2.4. Salinity Measurement

**Figure 13:**
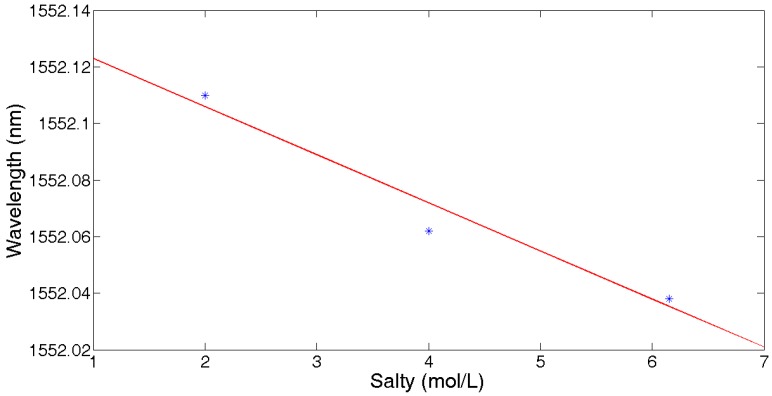
Central wavelength vs. salinity.

For salinity measurement, the UFBG4 was placed into a container with 30 ml deionized water, which means that the salinity was 0 mol/L. Then, 10.8 g NaCl was added to the water, bringing the concentration of saturated salt solution to 6.154 mol/L, and the central wavelength was shifted down to 1552.038 nm. Following this, deionized water was injected into the container to obtain a salinity of 4 mol/L and 2 mol/L, and the drift of the central wavelength was recorded, respectively. The linear fitting curve of the central wavelength with salinity changes is shown in Figure 13. It can be expressed as f(x)=−0.017x+1552.142, with the salinity sensitivity about ~−17 pm/mol·L^−1^.

### 3.3. Cross-Sensitivity Investigations and Thermal Compensation

As expected, the cross-sensitivity between the temperature and other parameters including RH, salinity, and hydrogen was observed. From Equation (1), it is clear that without temperature compensation, a correct multi-parameter measurement is not possible. Generally, apart from the temperature effect, the cross-sensitivity among the other parameters measures can be negligible. Since FBGs are intrinsically temperature sensors, it is natural to solve this issue by taking the measured temperature near the test points as the role of temperature compensation in this UFBG sensing array. 

Based on Equation (1), the central wavelength is influenced by the temperature change ΔT and the strain induced by the target parameter change ΔM of the transducer layer (fiber coating). The stress effects induced by temperature change are described as $\alpha_{M}-\alpha$. Then the superposition of the central wavelength shift $\lambda_{t}$ recorded by the interrogation system is given as:

(2) $$\begin{array}{l} \lambda_{t}=\lambda_{B}\cdot \left(1-P_{e}\right)\cdot \beta \cdot \frac{(b^{2}-a^{2})\cdot Y_{C}}{a^{2}Y_{F}+(b^{2}-a^{2})\cdot Y_{C}}\cdot M_{t}+\\ \lambda_{B}\cdot \left[\left(1-P_{e}\right)\cdot \frac{(b^{2}-a^{2})\cdot Y_{C}}{a^{2}Y_{F}+(b^{2}-a^{2})\cdot Y_{C}}\cdot \left(\alpha_{M}-\alpha \right)+\left(\alpha +\xi \right)\right]\cdot T_{t}+\\ \left\{\lambda_{0}-\lambda_{B}\cdot \left(1-P_{e}\right)\cdot \beta \cdot \frac{(b^{2}-a^{2})\cdot Y_{C}}{a^{2}Y_{F}+(b^{2}-a^{2})\cdot Y_{C}}\cdot M_{0}-\lambda_{B}\cdot \left[\left(1-P_{e}\right)\cdot \frac{(b^{2}-a^{2})\cdot Y_{C}}{a^{2}Y_{F}+(b^{2}-a^{2})\cdot Y_{C}}\cdot \left(\alpha_{M}-\alpha \right)+\left(\alpha +\xi \right)\right]\cdot T_{0}\right\} \end{array}$$

Where $\lambda_{0}$, $M_{0}$ and $T_{0}$ are the initial value of the central wavelength, target parameter, and temperature, respectively. $\lambda_{t}$ can be obtained by the interrogation system; $T_{t}$ can be calculated by UFBG3, the temperature sensor. 

**Figure 14:**
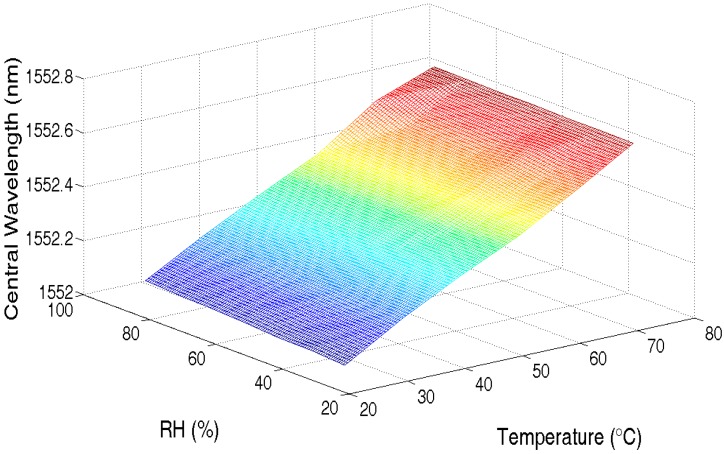
Central wavelength shift with both RH and temperature change.

Without loss of generality, the measurement of RH and temperature is taken as example. Sweeping the ambient temperature from 25 °C to 75 °C with a step of 10 °C and RH from 30% to 90% with a step of 10%, the central wavelength of UFBG2 was recorded from the interrogation system, as shown in Figure 14 after least-squares curve-fitting. 

## 4. Conclusions

An ultra-weak FBG array has been proved to be one of the most promising solutions for huge-capacity fiber sensing networks. In this work, we proposed and demonstrated a sensing array for multi-parameter measurements based on a UFBG with sensitive material coating. The central wavelength readout system employed uses two SOAs to separate the optical pulse from the sensors while rejecting unwanted signals caused by noise, crosstalk, and interference. This setup provides the advantage of testing various parameters in a single fiber without limiting temperature and strain measurement, and allows for a larger scale because of the strong multiplexing capability of UFBG.

This method was performed on a 144 UFBG sensors array with the reflectivity of UFBG ~0.04% for the four target parameters: hydrogen, RH, temperature, and salinity. The performance of multi-parameter sensing array was almost equal to result of the single-point FBG with general reflectivity. In order to solve the cross-sensitivity of multi-parameter sensors, with the temperature taking the primary effect to other parameters, a uniform solution was customized to divide the central wavelength shift caused by target parameters from that caused by the temperature effect. Based on the report of the multiplexing capacity of ultra-weak FBGs, it is expected that this scheme of UFBG-based sensing network can potentially multiplex thousands of multi-parameter sensors in single fiber, which shows promising prospects for the future.
